# Serum progesterone on the day of human chorionic gonadotropin (hCG) trigger as a predictor of in-vitro fertilization (IVF) outcome - a retrospective analysis of seven years

**DOI:** 10.5935/1518-0557.20220023

**Published:** 2023

**Authors:** Anshu Yadav, Nilofar Noor, Reeta Mahey, Neeta Singh, Vignesh Dwarakanathan, Neena Malhotra

**Affiliations:** 1 All India Institute of Medical Sciences, New Delhi, India

**Keywords:** progesterone, hCG trigger, live birth

## Abstract

**Objective:**

To investigate the relationship between progesterone (P4) levels on the day of hCG trigger and IVF outcomes.

**Methods:**

This is a retrospective analysis of IVF cycles from January-2013 to December-2019 from a single center. Women (21-39 years) submitted to IVF treatment for various infertility factors were included, while donor oocyte cycles and cancelled cycles were excluded from the study. The primary outcome measure was live birth rate.

**Results:**

A total of 2149 cycles were analyzed. Of these, 223 (10.38%) were in the low P4 group (<0.5 ng/ml), 1163 (54.12%) in the normal P4 group (0.5-1.5 ng/ml), and 763 (35.50%) in the high P4 group (>1.5ng/ml). The groups were comparable with respect to age, factor of infertility and baseline AMH. The antagonist protocol was significantly more prescribed to the high P4 group (p<0.001). Live birth rates were 14.4%, 21.6%, and 21% (p<0.001), respectively, in three groups. Univariate analysis found that total cetrotide dose, total number of retrieved and fertilized oocytes, total number of embryos formed, transferred, and vitrified, and P4 on the day of hCG (p<0.001) were statistically significant after adjusting for age and BMI. In multivariate logistic regression after adjusting for age and BMI, only high P4 (aOR:0.60; p<0.001), total cetrotide dose (aOR: 0.82; p<0.001), and total utilizable embryos (aOR:1.11; p=0.029) were statistically significant.

**Conclusions:**

Having an elevated serum progesterone level on the day of hCG trigger was associated with lower pregnancy rates, but this is still not a robust marker to predict live births. More good quality evidence is needed.

## INTRODUCTION

Progesterone (P4) is the primary hormone during the luteal phase of the menstrual cycle and is indispensable to prepare the endometrium for embryo implantation. In a natural cycle, fertilization and embryogenesis are synchronized with endometrial changes. Hence, when the blastocyst reaches the uterine cavity, conditions in the endometrium are perfect for implantation. The role of progesterone in favoring implantation in an estrogen primed endometrium goes beyond natural cycles to induced cycles during assisted reproductive technology (ART) treatment (Schoolcraft *et al*., 1991). Although the main function of progesterone is to support the endometrium in the luteal phase, fundamental research suggest of physiologic late follicular phase P4 increase, which, besides contributing to the timing of ovulation (Bosch *et al*., 2003), may be essential for follicular development (Xu *et al*., 2012).

Further, animal experiments have shown that blocking mid-cycle P4 production is detrimental to oocyte maturation (Labarta *et al*., 2011), oocyte fertilization competence (Al-Azemi *et al*., 2012) and granulosa/theca luteinization (Huang *et al*., 2016). While the early follicular phase progesterone is mainly of adrenal origin, in the late follicular phase progesterone is produced by the growing follicles that are synchronized before the LH surge. In an ovulatory cycle, serum progesterone concentrations are low during the early follicular phase and tend to increase 12-24 h before the onset of luteinizing hormone (LH) surge. Sometimes, premature LH surge leads to premature luteinization (PL) of the leading follicle, resulting in a rise in serum progesterone levels, an event seen more often in stimulated rather than natural cycles (Melo *et al*., 2006). This premature progesterone rise (PPR) occurs when progesterone levels rise above a threshold value near the end of the follicular phase or on the day of trigger in ART cycles (Venetis *et al*., 2013). Interestingly, this pre human chorionic gonadotropin (hCG) trigger progesterone increase also occurs on gonadotropin releasing hormone (GnRH) analogues, to keep serum LH levels under control (Vanni *et al*., 2017). The consequences of PPR are implantation failure and lower pregnancy rates (Huang *et al*., 2016). The proposed mechanisms underlying the detrimental effects of raised P4 include endometrial advancement due to raised P4 leading to embryo endometrium asynchrony, which is hostile for implantation and alters endometrial gene expression profile (Xu *et al*., 2012; Labarta *et al*., 2011; Al-Azemi *et al*., 2012).

The number of top-quality embryos was reduced with progesterone elevation (>2 ng/ml), suggesting it has deleterious effects on oocyte quality (Huang *et al*., 2016). But studies with oocyte donors have refuted this finding on oocyte quality in fresh and frozen-thawed transfer embryos (Melo *et al*., 2006; Venetis *et al*., 2013). Recently, embryo morphology was also found to be impaired in cases of raised progesterone regardless of intact blastulation rates (Vanni *et al*., 2017; Huang *et al*., 2016).

The effects of PPR on outcomes are conflicting, with studies, including a meta analysis, suggesting that PPR is associated with a lower probability of pregnancy during IVF cycles. (Felberbaum & Diedrich, 1999; Ozçakir *et al*., 2004; Hofmann *et al*., 1993) Contrary to this, some previous studies and a recent one did not find any significant differences in pregnancy rates during IVF with high or low progesterone levels on the day of ovulation trigger (Pangas *et al*., 2006; Hill *et al*., 2017). Large prospective studies including the Merit study (Hill *et al*., 2015) and a large retrospective cohort study (Oktem *et al*., 2017) supported that pregnancy rates were inversely related to progesterone levels on the day of trigger, especially when a threshold of 1.5 ng/ml was adopted. This threshold signifies the transition from follicular to luteal phase in the natural cycle (Werner *et al*., 2014), although it is still uncertain whether this threshold might be translated to stimulated cycles. The threshold value for freeze all in the previous studies was decided arbitrarily with no clear definition of the study population. Are all premature progesterone elevations detrimental or is it just an enhanced response? Should we go with a freeze all policy for all above this threshold of P4 in day of trigger, adding the burden of freezing to the cost of IVF cycle?

The clinical significance of increased follicular phase progesterone levels and its impact on pregnancy rates have been addressed, but conclusions are far from decisive. Although some postulate an adverse effect on ART outcome (Melo *et al*., 2006; Racca *et al*., 2018), others state that there is no significant effect on implantation or clinical pregnancy rates (Vanni *et al*., 2017; Bushaqer *et al*., 2018). Considering the ambiguity, this study was conducted with the objective to investigate the relationship between progesterone levels on the day of hCG trigger and IVF outcomes.

## MATERIALS AND METHODS

This study was conducted in the Reproductive Medicine Unit of our tertiary care referral hospital after ethical clearance from the Institute’s Ethics Committee (IEC-929/04.09.2020). A retrospective analysis of data of IVF cycles performed from January 2013 to December 2019 was done. Females aged between 21 and 39 years, who underwent IVF using agonist, antagonist, or micro-dose protocols were included in the study. The various indications for IVF treatment were tubal factor, endometriosis, male factor infertility, PCOS, and unexplained infertility. Cycles with donor oocytes and the ones that did not culminate with ovum pick up (cancelled cycles or cycles converted to intrauterine insemination) were excluded from the study. Cycles where a freeze-all policy for OHSS or agonist trigger was used were also excluded. A total of 2149 cycles were included in the study.

All patients underwent standard agonist, antagonist, or microdose protocols depending on their indication for IVF. In the agonist protocol, pituitary down-regulation was performed with 0.5 mg subcutaneous leuprolide (Zydus Cadila Healthcare Ltd.) starting from day 21 of the previous cycle. Fourteen days later, complete pituitary desensitization was confirmed by the detection of serum estradiol concentrations < 50 pg/ml, LH < 4 IU/l, no follicle with a diameter >8 mm, and endometrial thickness < 4 mm on ultrasound examination. The dose of leuprolide was reduced to half (0.25 mg) subcutaneously and gonadotropin (Recombinant FSH-Gonal F; Merck Serono, Mumbai, India) 150-375 IU/day was administered according to age, body mass index (BMI), and ovarian reserve. In the antagonist protocol, gonadotropin (Recombinant FSH-Gonal F; Merck Serono, Mumbai, India) 150-375 IU/day was administered according to age, BMI, and ovarian reserve. A transvaginal scan was performed on day 5 and the decision for starting the antagonist protocol was taken depending on ultrasound findings if one follicle >14mm, or E2 >500 pg/ml.

In microdose flare protocol injection, leuprolide 50 mcg twice a day was started from day 2 and gonadotropin (Recombinant FSH-Gonal F; Merck Serono, Mumbai, India) 150-450 IU/day from the next day with the dose depending on age, BMI, and ovarian reserve.

Serial follicle tracking was done to assess the ovarian response to stimulation and gonadotropin doses were adjusted accordingly. Additional gonadotropins were added. Human menopausal gonadotropin (HMG, Bharat Serum) was administered depending on response up to a maximum of 450IU/day. All patients were triggered with recombinant hCG (250 mcg, Ovidrel; Merck Serono, Mumbai, India) when there had at least 3 follicles ≥18 mm. Serum P4 was measured on the day of trigger with an in-house automated analyzer (Beckman Coulter Access 2, U.S.A) during the whole study period with a sensitivity of 0.05ng/ml and error <5%. Serial serum estradiol was measured in all cycles and serum LH levels analyzed in antagonist cycles at least on the day of adding antagonist. Serum estradiol and LH (in antagonist cycles) were also measured on the day of hCG trigger, using an in-house automated analyzer.

Transvaginal oocyte retrieval was performed 34-36 hours after the administration of the hCG trigger. Oocytes were fertilized either through conventional insemination or by intracytoplasmic sperm injection (ICSI) in patients with unexplained or male factor infertility. Fertilization was assessed 16-18 hours after IVF or ICSI. Up to a maximum of two good-quality embryos were transferred on day 3 or 5 under ultrasound guidance using a soft embryo transfer catheter (Cook’s medical Sydney, Australia). Excess embryos were cryopreserved. Decisions around elective freezing based on P4 levels were not followed among the cycles analyzed. Micronized Progesterone intramuscular injection 100 mg per day (Susten, Sun Pharma, India) was administered as luteal support from the day of oocyte retrieval. Pregnancy was confirmed by serum beta hCG estimation 16 days after embryo transfer. Ultrasound examination was performed 2 weeks after a positive beta hCG test to confirm fetal viability.

Primary outcomes measured per cycle were clinical pregnancy rate and live birth rate. The secondary outcomes measured were number of oocytes retrieved and fertilization rate, cleavage rate, embryo utilization rate (ratio of the sum of the number of embryos transferred and vitrified to the number fertilized oocytes). The fertilization rate was defined as the total number of fertilized oocytes by the total number of oocytes retrieved. Cleavage rate was defined as the total number of day 3 embryos by the total number of fertilized oocytes. Implantation rate was defined as the total number of gestational sacs visible on ultrasound by the total number of embryos transferred. The clinical pregnancy rate was defined as the presence of a gestational sac with a fetal pole and cardiac activity on transvaginal ultrasound at 6 weeks. The live birth rate was defined as the percentage of all cycles that lead to live births and is the pregnancy rate adjusted for miscarriages and stillbirths.

### Statistical analysis

Data analysis was carried out using Statistical package STATA version 12.0. Continuous variables were tested for normality assumptions using appropriate statistical tests. Descriptive measures such as mean and SD were reported for normally distributed data. Medians and interquartile ranges were reported for non-parametric data. All participants were categorized into three groups based on serum progesterone on the day of hCG trigger (P4) as low P4 <0.5 ng/ml, normal P4= 0.5-1.5 ng/ml, or high P4 >1.5 ng/ml. Actually, there is no standard cutoff for elevated premature P4. Different studies have taken different cutoff values varying from 0.8 to 1.5 ng/ml, depending on the progesterone assays used and the clinical outcomes. Therefore, we have chosen these arbitrary cutoff values to see how they might affect the outcomes. Outcome variables were compared within each category. Comparison of mean values within subgroups was carried out using Student’s T-test. Similarly, the comparison of median values within subgroups was compared using the non-parametric Mann-Whitney U test. Qualitative data were expressed as frequency and percent values. Categorical data were compared via the chi-squared test. A *p*-value of the trend was reported for estradiol level across the categories of ordinal variables. For all statistical tests, a two-sided probability of *p*<0.05 was deemed significant. We conducted logistic regression to look for the association between live birth and its predictors. First, the unadjusted odds ratio was calculated with live birth as the outcome and AFC (antral follicle count), periovulatory follicular count, estradiol and progesterone on the day of trigger, ET on the day of trigger, the total dose of FSH, total days of stimulation, total days of cetrotide, total oocytes retrieved, total embryos formed, total embryos vitrified, and total embryos transferred. Variables with a p-value less than 0.25 were considered for the adjusted model. For multivariable logistic regression, multicollinearity was tested using a variance inflation factor in Stata using the if command. Only one variable among the multi-collinear variables was retained in the multivariable logistic regression model. A cutoff value of 1.5 ng/ml for P4 levels on the day of trigger was taken to stratify patients into groups.

## RESULTS

A total of 2149 cycles were analyzed. Of these, 223 (10.38%) belonged to the low P4 group (<0.5 ng/ml), 1163 (54.12%) to the normal P4 group (0.5-1.5 ng/ml), and 763 (35.50%) to the high P4 group (>1.5 ng/ml) (Table 1).

**Table 1:** Number of IVF cycles

	**Serum progesterone on the day of hCG**			
	**Low**	**Normal**	**High**	**Total**
	**< 0.5 ng/ml**	**0.5 to 1.5ng/ml**	**> 1.5 ng/ml**	
Number of cycles n (%)	223 (10.38)	1163 (54.12)	763 (35.50)	2149 (100)

P4=Serum progesterone on the day of hCG trigger; a=Number (%)

The three groups were comparable for age and factor of infertility. Mean (±SD) BMI was 24.90 kg/m^2^ (±4.17), 25.03 kg/m^2^ (±3.82) and 24.79 kg/m^2^ (±7.96) in the low, normal, and high P4 groups, respectively (*p*=0.02). Use of an agonist protocol resulted in significantly more women falling into the normal P4 group; use of an antagonist protocol led to significantly more women in the high P4 group; and microdose flare led to significantly more women in the low P4 group (*p*<0.001). Mean (±SD) antral follicle count (AFC) was 13.9 (±6.5), 13.3 (±5.9), and 14.2 (±6.6) (*p*=0.007) in the in the low, normal, and high P4 groups, respectively. Mean AFC was significantly higher in the high P4 group. Mean (±SD) AMH was 3.94 (±2.61), 3.84 (±2.51), and 4.20 (±2.97) (*p*=0.062) in the low, normal, and high P4 groups, respectively. Mean (±SD) day 2 FSH was 6.63 (±4.59), 6.21 (±2.24), and 6.11 (±3.30) (*p*=0.022) and mean (±SD) day 2 LH was 5.40 (±3.70), 4.64 (±3.23), and 4.90 (±3.32) (*p*=0.007) in the low, normal, and high P4 groups, respectively. The mean (±SD) number of days of stimulation in the three groups were 10.52 (±2.34), 10.85 (±1.9), and 11.15 (±2.0) (*p*<0.001). The median total dose of FSH was 2950 (2100-3675) in the low P4 group, 3185 (2483.5-4050) in the normal P4 group, and 3300 (2525-4047.5) in the high P4 group (*p*<0.001). On the day of hCG trigger, serum estradiol levels were 2171 pg/ml (1294-3954), 3096 pg/ml (2008-4722), and 4425 pg/ml (2693-5060) in the three groups. Hence, serum estradiol was significantly greater in high P4 groups (*p*<0.001). The median number of follicles on the day of hCG trigger in the three groups was 7 (5-9), 7 (5-10), and 8 (6-11), respectively (*p*<0.001) (Table 2).

**Table 2:** Baseline variables

**Variable**	**Serum progesterone on the day of hCG**			
	**Low**	**Normal**	**High**	***p* value**
	**< 0.5 ng/ml**	**0.5 to 1.5 ng/ml**	**> 1.5 ng/ml**	
Age (µ±SD)	31.22±3.94	31.30±3.81	31.38±3.99	0.867
BMI (µ±SD)	24.90±4.17	25.03±3.82	24.79±7.96	0.02
Male factor n (%)	48 (21.52)	246 (21.15)	202 (19.92)	0.093
Female factor n (%)	167 (74.88)	856 (73.60)	579 (75.88)	0.911
Protocol n (%)				<0.001
Agonist	128 (59.0)	749 (64.85)	410 (54.38)	
Antagonist	69 (31.80)	343 (29.70)	304 (40.32)	
MDF	20 (9.22)	63 (5.45)	4 (5.31)	
AFC (µ±SD)	13.9±6.5	13.3±5.9	14.2±6.6	0.0069
AMH (µ±SD)	3.94±2.61	3.84±2.51	4.20±2.97	0.0616
Day 2 FSH µ±SD	6.63±4.59)	6.21±2.24)	6.11±3.30)	0.0216
Day 2 LH µ±SD	5.40±3.70	4.64±3.23	4.90±3.32	0.0065
Days of stimulation µ±SD	10.52±2.34	10.85±1.9	11.15±2.00	<0.001
Total dose of FSH [Median (Range)]	2950 (2100-3675)	3185 (2438.5-4050)	3300 (2525-4047.5)	<0.001
Follicles on day of hCG [Median (Range)]	7 (5-9)	7 (5-10)	8 (6-11)	<0.001
ET on day of hCG [Median (Range)]	8.9 (8-10)	9 (8-10)	8.9 (8-10)	0.080
E2 on day of hCG [Median (Range)]	2171 (1294-3954)	3096 (2008-4722)	4425 (2693-5060)	0.0001

BMI=Basal Metabolic Index; MDF=Micro dose flare; AFC=Antral follicle count; AMH=Anti Müllerian hormone; FSH=Follicle stimulating hormone; LH=Luteinizing hormone; hCG=Human chorionic gonadotropin; ET=Endometrial thickness; E2=Serum estrogen on the day of hCG trigger

The median number of oocytes retrieved per cycle in the three groups was 5 (3-8), 7 (4-11) and 8 (4-12) (*p*<0.001). Fertilization (78.3%, 73.6%, 71.7%; *p*=0.161) and cleavage rates (95.2%, 95.4%, 94.4%; *p*=0.935) were comparable in the three groups. The mean (±SD) number of grade 1 embryos was 3.05 (±2.18) in low P4 group, 3.91 (±3.00) in the normal P4 group, and 3.99 (±3.22) in the high P4 group (*p*<0.001). The mean (±SD) number of embryos transferred per cycle was 2.29 (±1.19) in low P4 group, 2.49 (±1.17) in the normal P4 group, and 2.37 (±1.35) in the high P4 group (*p*=0.042); and the mean number of embryos vitrified per cycle was 0.79 (1.78), 1.19 (2.49), and 1.38 (2.61) in the three groups (*p*<0.001). Embryo utilization rate was 65.6% in the low P4 group, 62.7% in the normal P4 group, and 60.9% in the high P4 group (*p*=0.370). Pregnancy rate was 30.0% (24.1%-36.5%) in the low P4 group, 28.5% (25.9%-31.2%) in the normal P4 group, and 20.7% (17.9%-23.7%) in the high P4 group (*p*<0.001). Live birth rate was 14.4%, 21.6%, and 21% (*p*<0.001) in the three groups, respectively (Table 3).

**Table 3:** Outcome variables

**Outcome per cycle**	**Serum progesterone on the day of hCG**			
	**Low**	**Normal**	**High**	***p* value**
	**< 0.5 ng/ml**	**0.5 to 1.5 ng/ml**	**> 1.5 ng/ml**	
Oocytes retrieved [Median (Range)]	5 (3-8)	7 (4-11)	8 (4-12)	<0.001
Fertilization rate (%)[Median (Range)]	78.3 (75.9-80.6)	72.5 (71.6-73.4)	71.7 (70.6-72.8)	0.161
Cleavage rate (%)[Median (Range)]	95.2 (93.7-96.5)	95.4 (94.8-98.9)	94.4 (93.7-95.1)	0.935
Grade 1 embryos µ±SD	3.05 (2.18)	3.91 (3.00)	3.99 (3.22)	0.0006
Embryos transferred µ±SD	2.29 (1.19)	2.49 (1.17)	2.37 (1.35)	0.042
Embryos vitrified µ±SD	0.79 (1.78)	1.19 (2.49)	1.38 (2.61)	<0.001
Embryo utilization rate (%)[Median (Range)]	65.6 (62.4-68.6)	62.7 (61.5-63.9)	60.9 (59.4-62.3)	0.370
Pregnancy rate (%)[Median (Range)]	30.0 (24.1-36.5)	28.5 (25.9-31.2)	20.7 (17.9-23.7)	<0.001
Live Birth Rate (%)	14.4	21.6	21	<0.001

Live birth univariate analysis found that total dose of cetrotide, total number of oocytes retrieved, the total number of fertilized oocytes, total number of embryos formed, number of embryos transferred, number of embryos vitrified, and P4 on the day of hCG (*p*<0.001) were found statistically significant after adjusting for age and BMI (Table 4). Due to multicollinearity between total number of oocytes retrieved, oocytes fertilized, embryos formed, embryos transferred, and embryos vitrified, we used only the total number of oocytes retrieved in the multivariate model. Therefore, multivariate logistic regression was carried out with these variables after adjusting for age and BMI. High levels of P4 (aOR:0.60 (95% CI: 0.47-0.77); *p*<0.001), total cetrotide dose (aOR: 0.82 (95% CI: 0.68-0.99); *p*<0.001) and total utilizable embryos (aOR:1.11 (95% CI: 1.07-1.16); *p*=0.029) were statistically significant.

**Table 4:** Univariate analysis of live birth outcome after adjusted for age and BMI

**Variables**	**Crude Odd’s Ratio**	**95% CI (LL-UL)**	**p value**
AFC	1.00	0.98-1.01	0.927
Periovulatory follicles	0.99	0.96-1.02	0.732
E2 on the day of hCG trigger	1.00	0.99-1.00	0.417
Total days of stimulation	1.00	0.95-1.05	0.893
Total dose of cetrotide	0.76	0.63-0.91	0.003
Total no. of oocytes retrieved	1.05	1.03-1.07	<0.001
Total number fertilized	1.10	1.07-1.13	<0.001
Total no. of embryos formed	1.11	1.08-1.14	<0.001
No. of embryos vitrified	1.08	1.03-1.12	<0.001
No. of embryos transferred	1.20	1.10-1.32	<0.001
Total dose of FSH	0.99	0.99-1.00	0.805
ET on the day of hCG	1.03	0.97-1.10	0.266
High P4 (>1.5 ng/ml) on the day of hCG	0.60	0.47-0.77	<0.001
Low P4 (<0.5 ng/ml) on the day of hCG	0.97	0.68-1.38	0.868

**Figure 1:**
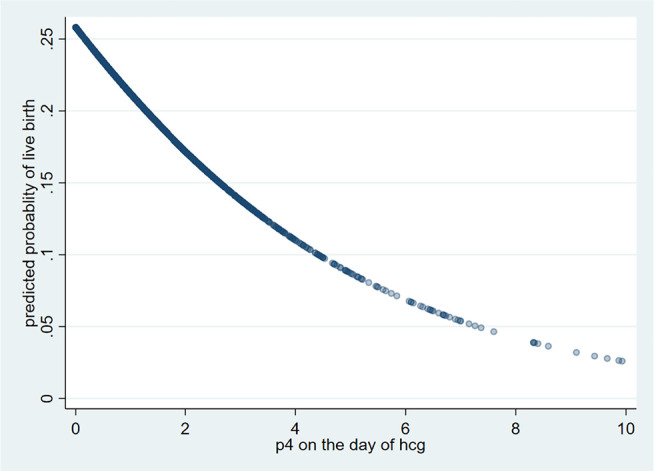
Predicted probability of live birth

**Figure 2:**
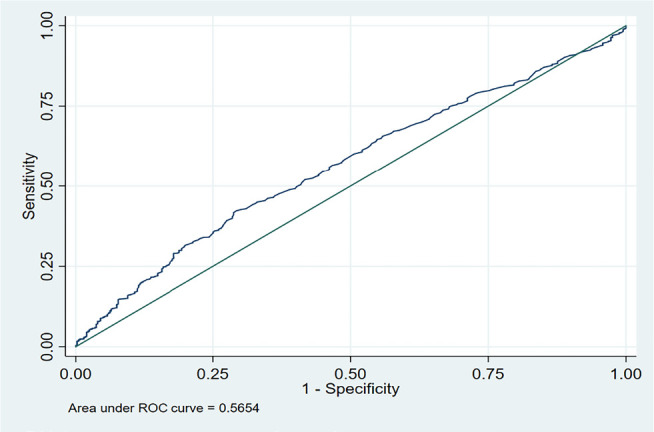
ROC curve

High P4 was associated with 40% lesser live birth odds compared to normal P4, and live birth odds increased by 11% with an increase of one utilizable embryo among our patients (Table 5). Probability of live birth as an outcome reduced with increase in progesterone on the day of hCG beyond the upper normal limit among our patients (Figure 1). ROC was made for progesterone level on the day of hCG for live birth rate. The area under the curve was 0.56, which suggests that progesterone is a poor marker to predict live birth (Figure 2).

**Table 5:** Multivariate logistic analysis after adjusting for age and BMI

**Variable**	**Adjusted Odd’s Ratio**	**95% CI (LL-UL)**	**p value**
High P4 (>1.5 ng/ml)	0.60	0.47-0.77	<0.001
Low P4 (<0.5 ng/ml)	0.97	0.68-1.38	0.868
Total cetrotide dose	0.82	0.68-0.99	0.045
Total utilizable embryos	1.11	1.07-1.16	<0.001

## DISCUSSION

As per our study, use of an agonist protocol resulted in significantly lower P4 levels, suggesting the better control of premature progesterone elevation by long-acting GnRH agonist as compared to an antagonist, which resulted in significantly more women in the high P4 group. The high P4 group had a lower predicted probability of live birth as compared to the low P4 group, and required a higher total dose of cetrotide. This group had a lower number of utilizable embryos, suggesting a detrimental effect of raised P4 above 1.5 ng/ml on oocyte quality.

The incidence of premature luteinizing hormone (LH) surge has been reduced to < 2% per cycle during controlled ovarian hyperstimulation (COH) with the use of gonadotropin-releasing hormone (GnRH) analogs for pituitary suppression (Felberbaum & Diedrich, 1999). Despite this achievement, PPR without any documented LH surge occurs in approximately 12-52% of cycles (Venetis *et al*., 2013). The etiopathogenesis of PPR in COH cycles is unclear, but various hypotheses have been proposed. Some authors advocate increased hormone receptor sensitivity due to higher cumulative exposure to estradiol and FSH (Bosch *et al*., 2003; Ozçakir *et al*., 2004), while others incriminate incomplete pituitary suppression by GnRH as a source of some LH secretion, which although insufficient to trigger ovulation, is enough to stimulate granulosa cells to produce progesterone (Hofmann *et al*., 1993). Another plausible explanation is the disruption of the oocyte granulosa cell regulatory loop (Pangas *et al*., 2006). Initial stimulation with a high FSH dose recruits a large number of growing follicles, resulting in increased ovarian steroidogenic activity and progesterone production (Bosch *et al*., 2003).

The association of follicle number with P4 levels has been documented in numerous large studies (Hill *et al*., 2015; 2017). They have demonstrated the cause of premature P4 elevation to be primarily due to an excessive number of follicles present, as noted in our study. Furthermore, FSH-only protocols and total FSH dose increase the risk of PPR (Oktem *et al*., 2017). This was also seen in our study, where longer stimulation and higher total dose of FSH (as seen in Group III) were associated with higher progesterone levels on the day of trigger. Conversely, the addition of LH to protocols decreased the risk of premature P4 elevation (Werner *et al*., 2014) by upregulating the aromatization of progesterone to estradiol (Hill *et al*., 2017).

A study (Bushaqer *et al*., 2018) compared the effect of elevated progesterone in agonist and antagonist cycles and concluded that clinical pregnancy rates were adversely affected only in antagonist cycles, although this was not replicated in other studies. Our study found no significant difference between clinical pregnancy rates with respect to different protocols used.

In a retrospective study (Racca *et al*., 2018) of 3400 antagonist ICSI cycles, the authors concluded that elevated progesterone was associated with decreased embryo utilization rates and fresh and cumulative live birth rates. A systematic review and meta-analysis (Venetis *et al*., 2013) of over 60,000 IVF cycles concluded that a premature P4 elevation was not related to oocyte quality, as there was no impact on donor-recipient cycle outcomes or subsequent frozen-thawed embryo transfers. Similarly, we found that elevated P4 levels did not seem to have a significant effect on embryo utilization rates, although they were associated with a significantly lower pregnancy rate.

More recent studies have strived to segregate the effects that PPR might have on the endometrium and on embryo quality (EQ). Even though it has been suggested that EQ is not affected by PPR (Melo *et al*., 2006; Xu *et al*., 2012), recent studies have postulated a detrimental effect (Huang *et al*., 2016; Vanni *et al*., 2017).

The authors of another recent study (Evans *et al*., 2018) found that in cycles affected by prematurely elevated P4 levels, vitrification of all embryos and performing a subsequent frozen embryo transfer yielded higher pregnancy rates. This was attributed to the detrimental effects of elevated P4 on the endometrium, resulting in endometrium-embryo asynchrony and implantation failure in fresh cycles.

As previously reiterated, there is no consensus regarding the effects of PPR on IVF outcomes. In a retrospective analysis of 2351 patients, Wu *et al*. (2019) concluded that progesterone elevation on the day of hCG trigger had a detrimental effect on the live birth rates of low and intermediate ovarian responders, but not in high responders. Furthermore, they deduced the critical value of progesterone levels on the day of hCG trigger to be 1.0 ng/mL in low responders and 2.0 ng/mL in intermediate responders. In a retrospective analysis of 2723 cycles, Santos-Ribeiro *et al*. (2014) found that live birth rates were significantly lower in patients with both low (<0.5 ng/ml) and high (>1.5 ng/ml) late follicular progesterone levels. We found the same relationship among those with high progesterone. But no significant reduction in live birth rates was found in the participants with low progesterone on the day of trigger, possibly due to the use of supplemented progesterone during the luteal phase. However, this explanation needs to be tested further.

Recommended interventions to avoid premature elevation of progesterone include close monitoring of serum progesterone levels and administering the hCG trigger before an excessive rise in progesterone (when progesterone levels reach 1 ng/ml to 1.2 ng/ml). Limiting the total dose of FSH and the use of a step-down protocol may also help to prevent premature progesterone elevation. Finally, if premature elevation does occur, then a freeze-all method should be considered (Bosch *et al*., 2003; Santos-Ribeiro *et al*., 2014).

The main limitation of our study is its retrospective nature. However, due to its large sample size, this study offers robust evidence regarding the detrimental effects of elevated progesterone on the day of trigger on pregnancy and live birth rates. The impact of raised P4 may not be a true reflection on the pregnancy outcome, since having a raised P4 on the day of trigger was a criterion to adopt a freeze-all strategy. Therefore, properly designed randomized trials are warranted to see the true impact of elevated progesterone on pregnancy outcomes.

## CONCLUSION

Elevated serum progesterone levels on the day of hCG trigger were associated with lower pregnancy rates. We still need robust evidence to derive a cutoff value for the adoption of a freeze-all strategy or some combined index including patient ovarian response and number of oocytes retrieved. Depending on how progesterone elevation impacts pregnancy outcomes, individualized cutoff values can be derived for patients considering ovarian response. More precise, sensitive, and standardized progesterone assays are needed.
